# Pooled Screening for Synergistic Interactions Subject to Blocking and Noise

**DOI:** 10.1371/journal.pone.0085864

**Published:** 2014-01-16

**Authors:** Kyle Li, Doina Precup, Theodore J. Perkins

**Affiliations:** 1 School of Computer Science, McGill University, Montreal, Quebec, Canada; 2 Ottawa Hospital Research Institute, Ottawa, Ontario, Canada; 3 Department of Biochemistry, Microbiology and Immunology, University of Ottawa, Ottawa, Ontario, Canada; University of Westminster, United Kingdom

## Abstract

The complex molecular networks in the cell can give rise to surprising interactions: gene deletions that are synthetically lethal, gene overexpressions that promote stemness or differentiation, synergistic drug interactions that heighten potency. Yet, the number of actual interactions is dwarfed by the number of potential interactions, and discovering them remains a major problem. Pooled screening, in which multiple factors are simultaneously tested for possible interactions, has the potential to increase the efficiency of searching for interactions among a large set of factors. However, pooling also carries with it the risk of masking genuine interactions due to antagonistic influence from other factors in the pool. Here, we explore several theoretical models of pooled screening, allowing for synergy and antagonism between factors, noisy measurements, and other forms of uncertainty. We investigate randomized sequential designs, deriving formulae for the expected number of tests that need to be performed to discover a synergistic interaction, and the optimal size of pools to test. We find that even in the presence of significant antagonistic interactions and testing noise, randomized pooled designs can significantly outperform exhaustive testing of all possible combinations. We also find that testing noise does not affect optimal pool size, and that mitigating noise by a selective approach to retesting outperforms naive replication of all tests. Finally, we show that a Bayesian approach can be used to handle uncertainty in problem parameters, such as the extent of synergistic and antagonistic interactions, resulting in schedules for adapting pool size during the course of testing.

## Introduction

The complex machinery of the cell is capable of producing strong, unexpected interactions between its individual components or other factors. A prime example of this is the phenomenon of synthetic lethality [1]. A pair of genes is synthetically lethal if the deletion of either gene individually has no or minimal influence on the organism, yet the deletion of both kills the organism. Networks of such interactions have been shown to contain important information about pathway and process relationships between genes [2], and so discovering these interactions is of great interest. Another important example is the Yamanaka factors, a set of four genes (Oct-3/4, SOX2, c-Myc and Klf4) whose overexpression can transform differentiated cells back into a pluripotent state very much like that of embryonic stem cells [3], [4]. This discovery has had numerous implications for stem cell research, including ready production of embryonic-like stem cells without the use of embryos, generation of patient-specific stem cells, and a greater understanding of the networks controlling stemness and differentiation more generally [5], [6]. Notably, none of the four factors are individually sufficient to restore a stem-like state, and indeed, Yamanaka and colleagues discovered the four factors by simultaneously overexpressing 24 known stem cell-related factors—a simple, though quite effective, pooling strategy [3]. Interactions are also important in the pharmaceutical world. While adverse interactions are a well-known clinical problem [7], interactions can also be beneficial. Multi-component therapies, which rely upon synergistic interactions between individually ineffective or weak drugs, are increasingly being used to address complex diseases such as cancer, HIV/AIDS, diabetes, and immune disorders [8]–[11].

Discovering interactions can be difficult. One reason is the sheer number of interactions that are possible. Abstractly, if we have 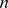 “factors” which may interact, then there are 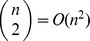 possible pairwise interactions, 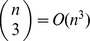 possible three-way interactions, and so on. Often, the number of actual interactions is vastly smaller than the number of potential interactions. For instance, in the largest screen for interactions between pairs of yeast genes to date [2], approximately 3% of the 5.4 million pairs tested showed a significantly unexpected influence on growth rate, and only a fraction of those were synthetically lethal. Similarly low rates of unexpected interactions have been observed in the relatively few attempts at high-throughput pooled drug screening [11]–[14]. Thus, exhaustive testing for interactions requires significant effort and has a rather low success rate.

Another source of difficulty is that interactions between factors may be masked by other factors, variously called blockers, inhibitors or antagonists [15]–[17]. In drug screening, the presence of one compound, which itself does not affect the biological target, may nevertheless neutralize the positive effect of compounds with which it is combined [17]. Blocking has also been identified as a challenge in screening DNA libraries [15], [18]. While we are not aware of genes whose expression blocks the reprogramming ability of the Yamanaka factors, it was recently shown that depleting Mbd3 greatly increases the efficacy of reprogramming—that is, the fraction of cells that return to a stem-like state [19]. Thus, Mbd3 is a strong, though not absolute, inhibitor of the Yamanaka factor synergy.

A further difficulty is that one always has to consider the possibility that a test may produce a false positive or false negative result (e.g. [20]–[24]). In high-throughput screens, both types of false results are common, and the experimental design must be able to account for such errors. A naive strategy is simply to replicate each test a fixed number of times, say 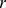. This allows one to gain greater certainty in the results, reducing the chance of both false positives and false negatives. However, this strategy increases the experimental burden by a factor of 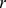, which is often considered prohibitive. An alternative, and probably more common strategy, is to perform an initial screen and then conduct confirmatory testing only on the positive results from the screen. This allows one to eliminate false positives from further consideration, but it does not address false negatives at all.

In principle, pooled screening offers ways to address all three of the difficulties just mentioned. To introduce the idea of pooled testing, let us consider the seminal work of Dorfman [25], who discussed the problem of testing blood samples of potential military recruits for signs of syphilis. The test for syphilis was very sensitive. For reasonable pool sizes 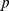 (meaning 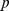 different blood samples are combined and then tested), a negative reading could be assumed to mean that none of the original samples were positive. However, a positive reading would mean that at least one of the original samples was positive. In this case, the individual samples would then need to be retested to identify precisely which recruits were infected. Dorfman showed that if the overall prevalence of syphilis is sufficiently small, so that relatively few pools are positive, then performing the pooled screen plus the positive-pool follow-ups can be far more efficient than testing each recruit's blood individually. Moreover, he showed how to select an optimal pool size based on the estimated prevalence of the disease.

Since Dorfman's work, the theory and practice of pooled screening has expanded enormously (see [14], [26], [27] for theory as well as pointers to many application areas). In the most standard formulations of screening problems, which omit synergy and antagonism, methods for dealing with testing errors range from simple grid-based schemes [27] to the recently-developed and powerful Shifted Transversal Design [11], [24], [28], [29]. A basic principle of such designs is that any individual factor appears in multiple pools, reducing the possibility of false negatives. Indeed, screens can be designed to automatically correct for a bounded number of testing errors (either false positives or false negatives) even without follow-up testing, giving a guaranteed degree of robustness (e.g., [15], [ 24], [ 28]).

Error-resilient schemes also provide some protection against antagonism. For instance, imagine a high-throughput drug screen in which there is a particular active compound 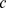. Compound 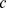 will be tested multiple times in combination with other compounds, and will fail to be detected only if every one of those pools contains an inhibitor. (These could be viewed as “false negatives”, though the tests are really correct, given the presence of the unknown inhibitors.) A better approach, however, is to employ a design that explicitly addresses the possibility of inhibitors [15], [16], [30]–[33]. Intuitively, given a bound on the number of inhibitors, such pooling designs ensure that the active compound(s) (or positive factors) occur in enough different pools with non-inhibiting factors that their effects will be detected.

The majority of the work on pooled screening does not address the issue of synergy or interactions between factors, although even single-factor schemes can be bent to this purpose. One can hold a factor 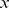 constant and search a library of other factors 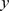 for interactions with 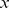 using a pooled screen. This approach has been used in screening DNA libraries [15] and yeast two-hyrbid screening [29], [34], to name two examples. There are, however, explicit schemes for searching for synergistic groups, sometimes called complexes, among a library of factors [12], [27], [35]. The essential problem is to create a screen ensuring that all combinations up to a certain size appear in one or more pools (depending on one's requirement for error tolerance). Relatedly, there is work on threshold-testing problems where it is not necessarily a particular combination of factors that produces a reading, but positive readings come when enough positive factors are included in a pool—and potentially, not too many inhibitors [36]–[38].

In this paper, we consider all three issues of synergy, antagonism and noisy testing. To our knowledge, the only previous works to address all three issues simultaneously are those of Chang *et al.* [32] and Chang *et al.* [33]. These works propose non-adaptive screening designs—that is, a way of selecting a set of pools given: the number of factors 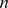, bounds on the number and sizes of synergistic groups, a bound on the number of inhibitors, and a bound on the number of errors (false positive or false negative) that will occur during the screen. After the screen is performed, the test results can be analyzed to identify all the synergistic groups correctly, without additional testing.

We focus instead on adaptive designs. In general, an adaptive design is a scheme for choosing a sequence of pools to test, in which the choice of next pool is allowed to depend on the outcomes of the earlier tests. Such designs can be quite sophisticated. We will, however, explore rather simple randomized designs, along the lines of Farach *et al.* [15]. In our view and experience, simplicity of a design is a point in favor of adoption. Moreover, the randomized designs we analyze lend themselves to analytical tractability. In particular, we are able to resolve questions such as: What is the expected screen size—the number of tests that need to be performed—to discover synergistic combinations of factors? What is the optimal pool size? How does noise in test readings require the design to be changed, and how does it affect sample size and optimal pool size? How can we design a screen if we do not know how many factors may be interacting or how many factors may be blocking an interaction?

Our analysis also differs from most previous work, and in particular Chang *et al.* [32] and Chang *et al.* [33], in the manner that testing errors are modeled. Most analyses assume an absolute bound 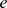 on the number of errors that will occur during a screen. While this is better than assuming no errors at all, we consider it unrealistic that the number of errors 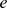 is independent of the size of the screen. Instead, we assume that each test has a fixed probability of producing an error. Under this assumption, no non-adaptive screening strategy can absolutely guarantee success—which is another motivation for our interest in adaptive designs. Conversely, even simple randomized adaptive screens can be guaranteed to eventually find synergistic groups with probability one, if they are allowed to proceed long enough.

Although our analysis is largely developed from the point of view of group testing theory, pooled screening problems can also be related to the theory of learning sparse Boolean functions [39]–[47]. In a generic version of this problem, we assume the existence of a Boolean function of interest 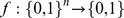. However, we assume that 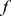 really only depends on 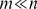 of the input variables. Identifying those 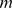 relevant inputs is therefore of great interest—sometimes of greater interest than identifying exactly how 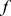 depends on those inputs. We can relate sparse Boolean functions to the pooled screening context by saying that the 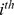 input feature is 1 if the 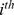 factor is included in the pool, and 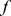 just returns whether or not the pool tests as positive. In the standard pooled testing problem, without synergy and without blockers, a pool is supposed to read positive if any of 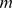 individually-positive factors is present in the pool. Thus, 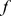 is simply the disjunction of the 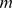 corresponding input features. In a formulation that allows for synergy, and assuming for simplicity that we seek a single 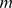-way synergy, 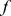 is instead the conjunction of the 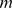 corresponding input features. If we additionally allow for 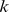 blockers, then 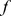 would be the conjunction of the 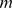 synergy features and the negation of the 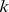 blocking features. In the present study, we will generally assume that 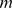 is small compared to 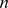; however, we will make no such assumption about 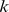. As such, our problem does not technically fit within the assumptions of a sparse Boolean function learning problem. Nevertheless, as has been shown for sparse Boolean function learning [41], [42], [46]–[48], we will see that relatively few pools—even if selected randomly—are sufficient for identifying the 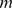 factors of interest.

## Results

### Randomized pooled screening guarantees discovery of synergistic combinations, despite blocking, and can vastly outperform exhaustive testing

We begin by analyzing a basic scenario with synergy and blockers. We assume there are 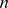 individual factors in which we are interested—genes, drug compounds, etc.—and that we can test these factors either individually or in combinations. Each test results in either a positive or a negative outcome. A positive outcome is the outcome of interest—for instance, synthetic lethality between two genes, or synergy between drugs. A negative outcome means the factor or combination of factors tested either had no effect or merely had the expected effect, and is therefore not of interest. As mentioned above, in screens for gene interactions related to yeast growth rates, just a few percent of potential interactions turn out to be real, and strong interactions are rarer yet. Similarly, in a typical drug screen, a few percent of individual compounds may have some effect on a biological target, while the number of synergistic combinations is expected to be quite small. Here, we make the pessimistic assumption that the set of 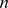 factors contains just a single synergistic combination of 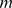 factors that has a positive effect. Therefore, the goal of the screen is to find this particular combination. We call these factors the *desirable factors*. Establishing the utility and feasibility of pooled screening under this scenario implies that it would be all the more useful under less pessimistic conditions. In the Discussion section, we outline how our results can straightforwardly be extended to the case of multiple synergistic combinations. We also assume that there are 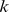 factors that are *blockers*. Whenever one of the blockers is present in a pool along with the desired factors, the positive effect is completely abolished. The remaining 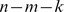 factors are *neutral*, and do not effect the outcome of the test. For now, we will assume that the parameters 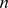, 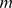, and 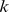 are all known—although of course the identities of the 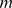 desirables and 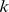 blockers are unknown. Later, we will lift this assumption, treating 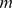 and 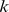 as unknowns about which we maintain probabilistic beliefs that can change during the course of testing. We will focus first on the noise-free case, in which a pool of factors gives a positive reading if and only if it contains all 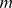 desired compounds, none of the 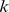 blockers, and any number of neutral factors.

The number of factors, 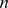, may be large—hundreds or thousands for genes, and possibly into the millions for large drug screens. We expect 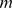 to be relatively small, as in the examples of synthetic lethality (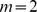), the Yamanaka factors (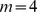), or drug cocktails (typically 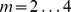). We also allow 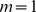, an individually-active factor, as a special case, although our primary interest is in identifying synergies between factors. The number of blockers, 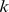, can vary widely.

**Figure 1:**
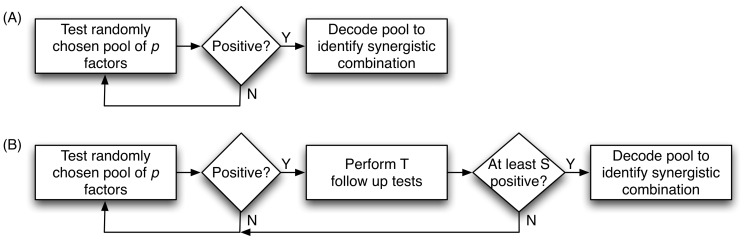
Sequential designs for pooled screening, in the case of noiseless tests (A), and with the possibility of noisy tests (B).

Pooled experiment designs often have two phases. In the first phase, a collection of pre-chosen pools are tested. In the second phase, sometimes called the *decoding* phase, members of positive pools are tested further, either individually or in groups, to determine the cause of the positive reading. In the first phase, the main design choices concern the sizes of the pools and the method used to assemble each pool. For our initial model of the screening problem, we consider the screening design shown in Figure 1A. Pools of size 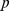 are drawn repeatedly, uniformly randomly, and with replacement, from the 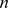 factors. This sequence of pools is tested until a positive reading is achieved. Recent work on drug screening has shown that a biased random selection procedure, which aims to better cover a feature space of chemical descriptors, can improve the efficiency of such randomized screening [13]. However, for simplicity and analytical tractability, we focus on the more straightforward selection method. For the screening procedure in Figure 1A, as long as random pools of size 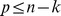 are chosen, the procedure will eventually find a positive pool with probability one—simply because there is at least one positive pool of this size. Thus, the first phase terminates with probability one.

Once a positive reading is obtained, the second phase of the design is responsible for identifying the desirable factors from the positive pool. There are 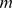 desirables that need to be discovered in the pool of size 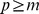. A simple approach, requiring 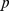 tests, is to exclude each factor from the pool in turn and test the rest of the factors as a pool. If the reading is negative, then the factor that was excluded is one of the 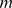 desired factors. If the reading is positive, then the factor that was excluded is neutral and can be discarded from the pool. More generally, because the positive pool cannot contain blockers, one could use any scheme for identifying 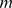-way synergies from a library of factors [12], [26], [27]—but in this case from just the 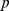 factors in the positive pool, rather than the entire library of 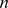 factors.

In the classic work of Dorfman [25], the optimal pool size is determined by trading off the costs of the first and second phases. In his formulation, larger pools increase the efficiency of the first phase, but tend to decrease the efficiency of the second phase, as more pools will be positive and their larger size means that more follow-up tests will be needed. In our study, the 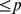 tests required by the decoding phase are generally inconsequential compared to the number of tests needed in the first phase. So, the efficiency of the first phase is our primary concern. Larger pool sizes are favored by the desire to reduce the number of tests needed. In particular, larger pools test more factors simultaneously and test more possible synergies simultaneously. However, the presence of blockers favors smaller pools, so that positive readings are not masked. This tension determines the optimal pool size. In some cases, particularly when the number of blockers 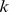 is very small, the second phase of the design can take substantial testing effort compared to the first phase, and should not be ignored. We return to this issue in the Discussion section.

We begin by computing the expected time (i.e., number of tests) until a positive reading is found in the first phase. In order to get a positive reading for a particular test, we must assemble a pool that contains all 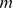 desirable factors, 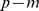 neutral factors, and no blockers. The total number of pools of this sort is 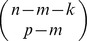, out of a total of 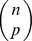 possible pools of size 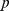. Thus, the probability that a randomly selected pool of size 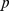 gives a positive reading is
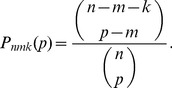
(1)As we have assumed that pools are drawn independently and tested sequentially until a positive reading is found, the time until obtaining a positive reading is just a geometric waiting time random variable, with success probability 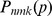. Therefore, the expected number of tests as a function of 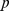 is
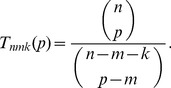
(2)


**Figure 2:**
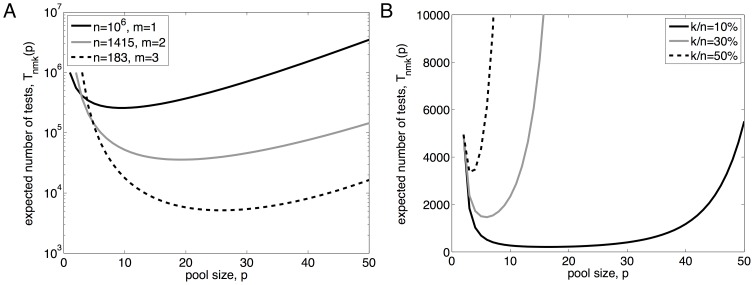
Expected number of tests in phase one, assuming noiseless tests, as a function of pool size, 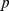. (A) Three different choices of library size, 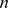, and synergistic group size, 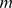, for which exhaustive combinatorial testing requires one million tests (or 500000 expected tests, assuming early stopping is allowed). In each case, the number of blockers is 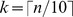, or approximately 10% of the library. (B) Varying 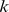, with 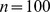 and 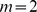.

To give some intuition for the relationship between 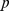 and 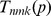, Figure 2 shows 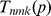 for varying values of 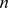, 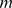, 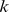 and 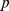. In panel A, the three curves show 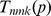 for the three cases: seeking a single active factor from a set of 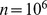, seeking a synergistically active pair of factors from a set of 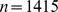 candidates, and seeking a synergistically active trio of factors from a set of 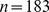 candidates. In each case, we assume 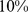 of the factors are blockers. These values were chosen because the straightforward approach of testing all 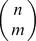 subsets takes approximately one millions tests in each case—a feasible number by current, high-throughput methods. If we imagine running through those one million tests, but stopping as soon as the desirable combination is identified, then the *expected* testing effort of the naive, exhaustive screen is 500000 tests.

An immediate observation is that pooled testing can greatly increase the efficiency with which the desirable factor(s) are identified. For the 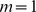 case, testing at the optimal pool size of 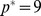 reduces the expected number of tests needed to 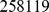
—a twofold reduction in testing effort. For 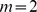 and 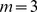, the optimal pool sizes of 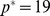 and 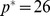 reduce the expected number of tests to 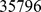 and 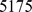 respectively, corresponding to fourteen-fold and nearly 100-fold reductions in testing effort respectively. Achieving these improvements requires relatively large pool sizes. Pool sizes as large as 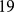 or 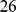 are feasible for gene overexpression or knockdown studies (as in [3]). They may or may not be feasible in drug screening, due to general toxicity effects; the study of Severyn *et al.* [11] used a pool size of ten. In any given case, there may be limits on how large a value of 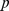 is feasible. However, even if one sticks to a relatively modest 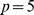, the expected numbers of tests needed for the cases 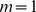, 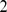, and 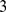 go down to 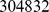, 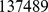 and 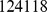 respectively—a savings of 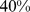 to 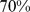. In this simple scenario, then, we see that pooled testing has the potential for greatly increasing the efficiency of discovering desirable factors or combinations of factors.

Figure 2B shows the effect of varying the number of blockers, 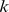, for 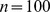 and 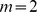. Increasing 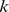 has a dramatic effect on the expected number of tests as well as the optimal pool size. Qualitatively, both panels show that 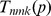 appears to initially decrease with 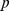 and then increase. Intuitively, 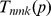 decreases at small 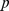 because of the increased chance of including all the desirable factors in the pool, but it increases at larger 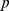 because of the increased chance of including blockers. (Shortly, we will show analytically that 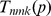 is unimodal in all but a few degenerate situations.) This implies that there is usually a single, optimal choice of 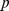, although the curves also show that there can be a significant range of values of 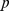 for which 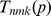 is nearly as good as at the optimal pool size.

Finally, we note that if one takes 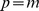, the minimum pool size with which it is possible to discover an 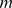-way synergy, the expected number of tests is just 
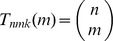
. This is exactly the same as exhaustively running through all possible 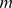-way combinations—although it is twice the expected effort, if we assume that the exhaustive screen ends when the combination is found.

To determine the optimal pool size for given 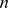, 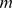 and 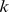, consider the ratio
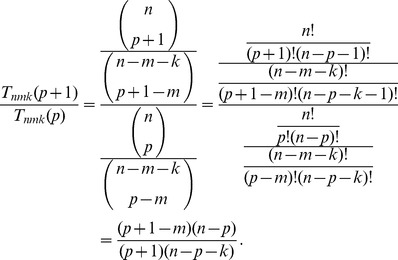
(3)


If this ratio is less than one, then testing with pool size 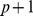 is more efficient than testing with pool size 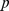. This leads to the following criterion.
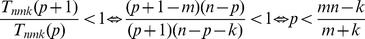
(4)


This confirms the observation (see Figure 2) that 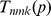 is unimodal in 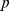, decreasing as 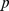 increases to a certain threshold and then increasing afterwards. If 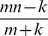 is an integer, then 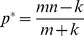 is the optimal choice of 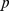. If 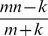 is not an integer, then the smallest integer greater than that, denoted 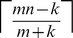 is the optimal choice. In either case, we can write the optimal choice for 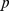 as
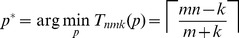
(5)


Equation 5 always yields a valid choice for 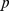 that falls in the range 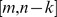 (see Materials and Methods for proof). This ensures termination of the screening procedure with probability one. It is possible that 
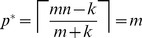
, so that the optimal pool size is no larger than the minimum necessary to discover an 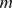-way synergy. In particular, this can happen when the number of blockers, 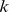, is very large. For example, in the case 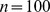 and 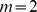, the optimal pool size is 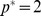 when 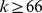. In such cases, 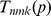 is monotonically increasing in 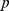. The case 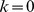 is a degenerate situation in which 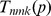 is monotonically decreasing in 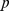 and 
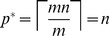
. As stated above, optimal pool size for us is determined by a tradeoff between library and synergy size, favoring large pools, and the number of blockers, favoring small pools. In the absence of blockers, there is really no need for phase one—the primary purpose of which can be viewed as identifying a pool or sub-library that still contains the synergistic group, but without any blockers. In this case, approaches for finding synergistic combinations without blockers should be employed [35].

**Figure 3:**
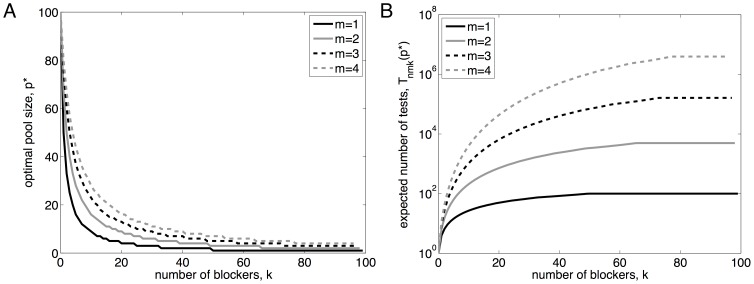
Optimal pool size and testing complexity. (A) The optimal pool size, 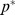, as a function of the number of blockers, 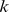, for varying numbers of desirable factors, 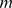. (B) The corresponding expected number of tests needed to identify the desirable factors. These curves are for library size 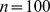.

Figure 3A shows 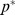 as a function of 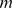 and 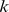, for 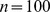. Optimal pool size drops rapidly at low 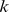, and plateaus at 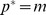 for high values of 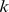. Figure 3B shows the expected number of tests for various 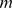 and 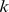 if the optimal pool size is used. The qualitative shape of the curves for 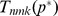 can be understood by analyzing the ratio 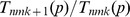.
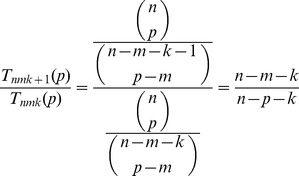
(6)For fixed 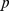, 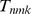 is non-decreasing in 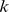, because 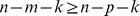. The ratio is greatest when 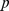 is large. If we consider 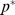 instead of a fixed 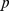, then intuitively, 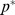 is largest when 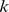 is small, so we expect 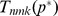 to be increasing fastest (in terms of ratio) when 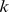 is small and to level off at large 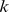, when 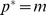.

### Testing errors do not change optimal pool size, and are best handled by follow-up testing on positive pools

In realistic situations, test results, especially from high throughput methods, may be erroneous. In such a scenario, a positive reading no longer guarantees that we have the desired combination in the pool. Similarly, a negative reading does not imply that we failed to have the desired combination and no blockers in the pool. In this section, we assume that the test has a fixed probability 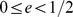 of producing an erroneous reading, either a false positive or a false negative. We assume the same error rate for both positive and negative readings for simplicity, but the case of different error rates is a straightforward extension.

In this new setting, a positive reading arises either from a truly positive pool with a correct reading (true positive) or from a truly negative pool with an incorrect reading (false positive). Let 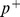 be the event that a randomly drawn pool is truly positive, 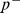 be the event that a randomly drawn pool is truly negative, and 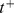 the event that a randomly drawn pool tests as positive. Then, the probability of getting a positive reading for a randomly chosen pool of size 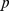 is
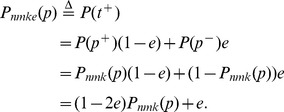
(7)Note that 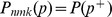 is the probability of drawing a truly positive pool, just as in the previous section.

To accommodate for testing errors, one generally uses replicates to maintain a certain level of confidence. There are many ways to incorporate replicates. The most straightforward scheme is to repeat every test some fixed number of times. In a subsequent phase, tests that are “sufficiently positive” are followed up. (Sufficiently positive may mean that all or most of the replicates test positive, though there are other ways to combine multiple tests, depending on the setting.) In the present setting, however, it turns out that replicating the tests on every pool is inefficient. Because the vast majority of tests are expected to be negative, replicating all those tests to be sure that they are negative wastes significant effort.

We propose an alternative, as shown in Figure 1B. We start with random pool selection and repeat until we obtain a first positive reading. This pool could be a false positive, so we perform 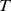 replicate tests on the same pool. A confidence level 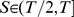 is defined such that if there are at least 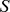 positive readings out of the 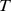 replicates, we consider the pool to be a true positive, and we proceed to the decoding phase (whose testing effort we ignore, as it is generally small). If, however, the 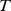 replicates do not contain 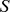 positive readings, we consider that the initial positive reading was a false positive, and we continue testing randomly drawn pools. Note that, in this scheme, it is possible that a truly positive pool is read as negative. In this case, the procedure simply continues to draw random pools of factors for testing. Although an occurrence of a positive pool may be “missed”, eventually, the procedure should draw a truly positive pool that tests as positive, both initially and in at least 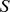 of the 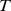 confirmation tests.

The expected number of tests required by this procedure obeys
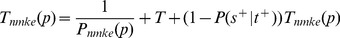
(8)where 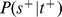 is the probability that a pool that tests positive in its first test also tests positive in at least 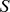 of the 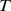 follow-up tests—that is, it is confirmed as a truly positive pool. The rationale behind this recurrence is simply that the total expected number of tests can be decomposed into the number of tests until obtaining the first positive reading, the automatic 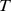 follow-up tests, and, if the follow-up tests do not confirm that the pool is a true positive, the expected tests from then on (which, as the process is memoryless, is the same as at the start of the process). The expected number of tests is thus
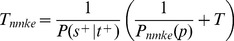
(9)


We will shortly consider the behavior of 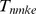 for various parameter values, but first let us derive the optimal pool size. The probability 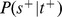 depends on the pool size, even though the notation does not make it explicit. Thus, the above formula for 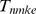 depends on 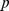 in two different places. Remarkably, it turns out that the optimal pool size is the same as for the noise-free case. In fact, that same pool size minimizes both the 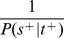 term and the 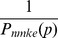 term simultaneously. The latter claim follows readily from Equation 7, keeping in mind that 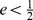, so that 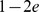 is positive. The claim that the same 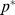 also minimizes 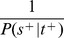 takes more effort to show. We relegate its proof to the Materials and Methods.

**Figure 4:**
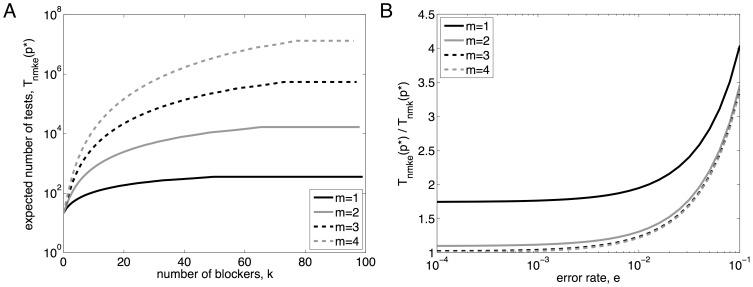
Number of tests needed, if one allows for the possibility of noisy test outcomes. (A) The expected number of tests as a function of the number of blockers, 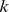, and the number of desirable factors, 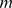, for 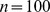 and noise level 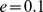, assuming screening with optimal pool size. (B) The ratio of the number of tests expected under the noisy model to the number expected under the noiseless model, for varying error rates 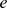, with 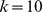.

Figure 4A shows the expected number of tests under the noisy-testing model, for 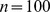, 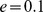, 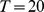, 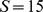, and varying 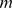 and 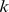, assuming the optimal pool size is used. With these choices of 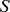 and 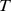, if a pool initially tests positive but is actually a negative pool, the chance of erroneously confirming it as a positive pool is approximately 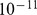. The chance of failing to confirm a truly positive pool is approximately 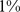; if this did happen, of course, the procedure would continue to look for a positive pool. Hence, with very high probability, the procedure finishes by identifying a truly positive pool. Qualitatively, the expected number of tests is very similar to the expectation under the noise-free model (see Figure 3B). Figure 4B shows the ratio of the expected number of tests under the noisy model to the expectation under the noise-free model, for varying error rates, 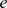, 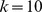, and with all other parameters the same. Naturally, when the error rate is small, little extra effort is involved. In the case 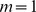, there is about 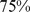 extra testing effort, which is almost wholly due to the 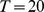 follow up tests. As the error rate increases, so does the testing effort ratio, reaching a value between three and four for these parameter settings. A more standard approach to dealing with the possibility of noisy readings is to replicate each test some fixed number of times, usually at least three. Even at a high level of noise, 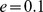, the testing scheme we propose is approximately as efficient as naive triplicate testing. Further, our scheme has the added benefit of producing a correct answer with very high probability. In contrast, the naive replication scheme has a nontrivial chance of missing the desirable combination, and is almost certain to return a large number of false positives. For instance, if we require all three replicates to be positive, then the truly synergistic combination has 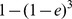 chance of being called positive. With 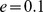, there is a 27% chance that it will not be correctly identified. At the same time, the expected number of false positives is 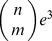. For instance, with 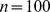, 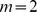, and 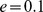 we expect 5 false positives, and with 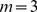 we expect 162 false positives. If we would require only 2 of 3 replicates to test positive, our chance of detecting the truly synergistic combination increases, but so does the expected number of false positives.

### Bayesian adaptive scheduling when the numbers of synergistic and/or blocking factors are unknown

So far, we have assumed that all the parameters of the problem (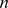, 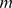, 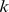 and 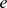) are known. In reality, this is usually not the case. Of course, the total number of factors 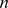, is typically known. Often, the error rate of the assay, 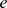, has been established based on calibration testing or previous screens. The value of 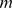 may well be unknown, though it is generally expected to be relatively small, say between 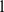 and 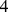. We expect that the number of blockers, 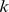, will often be unknown and that we can expect significant uncertainty about its value.

With unknown 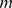 and/or 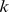, there are several ways to proceed. We could optimistically assume that 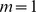 and 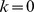. However, if our assumption is violated, we may find ourselves with an endless stream of negative results and no way to explain them. On the other hand, we could pessimistically assume that 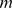 is large (say, four) and that 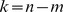. In this case, we would only test pools of size 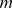, but this approach misses out on much potential gain in efficiency by using larger pools. Furthermore, such a situation is not what we expect in practice. We are left, then, with 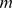 likely being small and 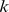 being somewhere between 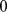 and 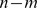.

In this section, we explore a Bayesian approach to handling uncertainty about the true values of 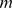 and 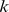. We assume that before screening begins, there is a prior belief 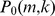 that represents our estimate of the chance that there are 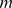 desirable factors and 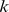 blockers. As we will show shortly, these beliefs can be updated as screening proceeds. For simplicity, we assume noise-free testing 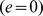, although the following can be generalized to the noisy testing case. With noise-free testing, if we obtain a positive pool, then we move to the decoding phase and our belief over 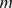 and 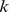 becomes irrelevant. As long as we continue to get negative readings, however, we can update our belief about 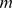 and 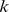.

Suppose that for the 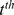 test, we choose a random pool of factors of size 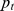. Below we describe several possible schemes for choosing the pool sizes as a function of 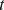. Let 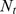 denote the event that the first 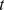 tests come out negative. Let 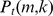 denote our belief over 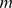 and 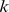 after 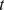 negative tests. The following equation describes how we can update the belief as each negative test result comes in. (Again, if we get a positive test, then we can discard our beliefs and proceed to the decoding phase.)
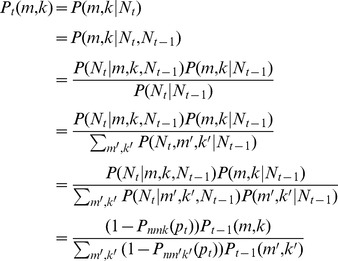
(10)


Because all the terms on the right hand side are known, this shows how the beliefs at time 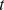 depend on the beliefs at time 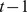.

This leaves the question of how to choose the sizes of pools to test. For given 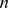, 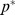 can be viewed as a function of 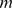 and 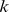. As 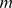 and 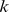 are unknown, we propose the expedient of choosing 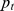 based on our beliefs after the first 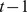 tests, 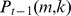. Specifically, we propose the pool size should be chosen by averaging the optimal pool size over the unknown parameters, rounding as necessary.
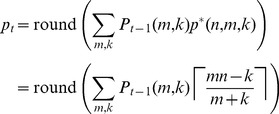
(11)Certainly, other choices are possible. For example, we might choose 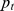 to minimize the expected number of tests, assuming no further change in pool size.
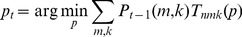
(12)Or, we might choose 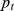 based on the expected values of 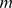 and 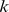, or some percentiles of their distribution. We leave a detailed exploration of such strategies for future work, and limit our attention to Equation 11 for determining pool sizes.

**Figure 5:**
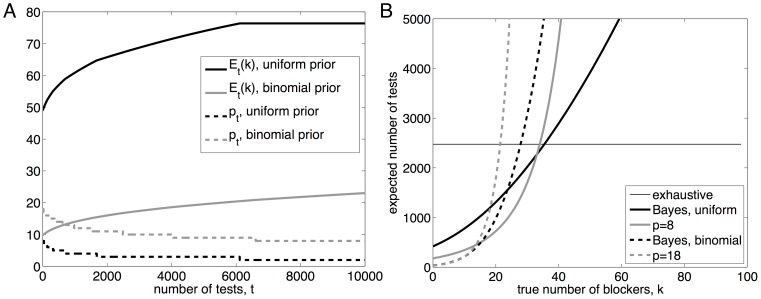
Pooled screening with uncertainty in the number of blockers. (A) The evolution of the expected value of 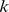 with respect to the belief over 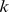, along with the pool size schedule 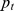, as testing proceeds. Repeated negative tests increase the belief that 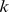 is larger, driving down the recommended pool size. (B) The expected number of tests until discovering the desirable combination of factors, for several fixed pool-sizes and for the Bayesian adaptive strategy. These results assume a set of 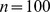 factors from which we seek a synergistic pair (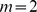).“Uniform” refers to the assumption that the belief about 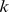 is initially uniform on the range 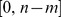.“Binomial” refers to the assumption that the initial belief about 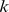 is binomial(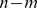, 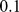).

As an example, suppose that 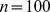 and that we know 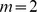, but that the number of blockers is uncertain. Figure 5A plots the sequence 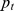 in two different cases. For one, we assume a uniform initial belief over 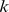: 
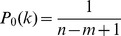
 for 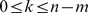. For the other case, we assume that each non-desirable factor has a 10% chance of being a blocker, leading to a binomial belief, 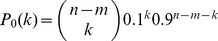. The figure also shows 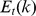, the expected value of 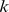 according to the belief distributions, as testing progresses. The figure shows that, in either case, the expected value of 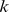 increases as testing progresses. Intuitively, this is because a large number of negative test results are more likely if 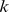 is higher. As a result, the suggested pool size drops as testing progresses, because larger (estimated) values of 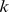 favor smaller pool sizes. Formally, this can be derived readily from Equation 10, using the fact that 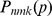 is non-increasing in 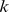. For the uniform initial belief, 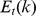 stops changing shortly after the 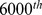 trial. This is because 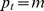 at the point. In this case, the probability of a positive test, 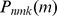, is the same regardless of 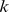, so we gain no new information about the value of 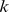.

Figure 5B shows the expected number of tests for each of these pool-size sequences, for different possible true values of 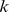. The horizontal line shows the expected number of tests under a naive exhaustive screen, which for 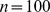 and 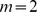 involves 
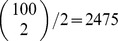
 tests. The thicker solid curve shows the number of tests taken by the pool-size schedule induced by a uniform belief for 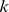. If 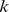 truly is small, this strategy requires less than 20% as much testing as the naive strategy, and there are some savings even if the true 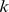 is as large as 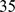. The dotted line shows the tests expected under the fixed choice 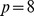, the initial pool size suggested by a uniform belief for 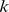. For 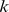 up to 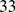 it actually does better than the adaptive Bayesian strategy, because it persists in using the larger, beneficial pool size 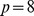, whereas the Bayesian strategy switches to a lower pool size if testing runs long enough. However, for large values of 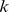, the Bayesian strategy does better by a large margin, because it correctly deduces the benefit of the smaller pool size. The dashed lines show the expected number of tests using the Bayesian strategy with the binomial initial belief over 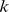, as well as the fixed strategy 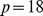, which is the optimal pool size for the initial belief. If the true 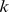 is close to what is predicted by the binomial belief, then these strategies perform dramatically better than the naive screen, and modestly better than the uniform prior. On the other hand, if the initial belief is far from correct, then these strategies perform disastrously, because they persist in using a pool size that is far too large. As with the uniform initial belief, the fixed choice 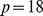 outperforms the Bayesian adaptive strategy for the smallest values of 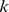 (though the difference is slight), and the adaptive strategy does better for larger values of 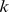.

**Figure 6:**
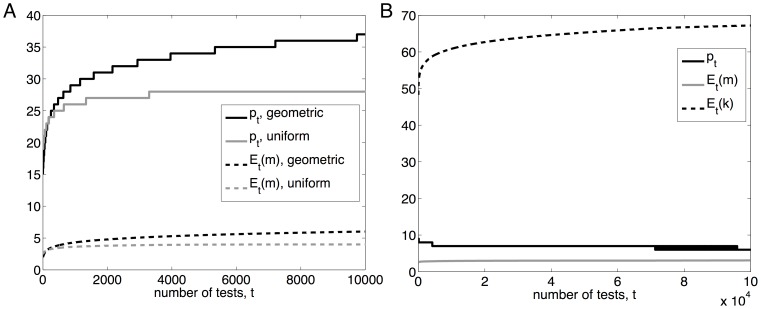
Pooled screening with uncertainty in the number of desirable factors and blockers. (A) When the number of blockers, 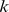, is known but the number of desirable factors, 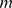, is not known, increasing numbers of negative tests are evidence for higher values of 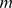, which raises the optimal pool size. (B) The situation in which both 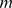 and 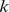 are unknown. Beliefs for both 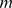 and 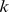 shift towards larger values over time (though it is difficult to see the increase in 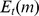 on this scale), resulting in competing forces on the ideal pool size.

In Figure 6A, we consider the case that 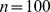 and 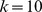, but that 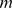 is unknown. We explore two cases for the initial belief over 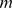: a uniform distribution on 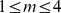, and a geometric distribution: 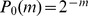. The figure shows that, as testing proceeds, the belief distributions for 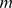 shift towards higher values, as higher values of 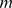 are a more likely cause of large numbers of negative tests. In this case, the pool size increases during the course of testing, as larger values of 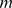 imply larger optimal pool sizes. In Figure 6B, we consider the case that 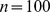 but neither 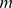 nor 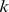 are known with certainty. We assume an initial belief that is uniform over all combinations of 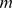 and 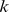 with 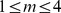 and 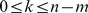. As the number of tests increases, the belief distributions for both 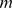 and 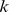 shift towards larger values. The increase for 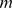 is difficult to see in the figure, but the expected value changes from approximately 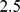 to 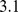 over the course of 100000 negative tests. The increasing estimates of 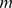 and 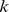 place competing forces on 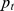. The former tends to increase the optimal pool size while the latter tends to decrease it. For the particular choices made here regarding 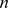 and the initial belief, the pool size turns out to decrease until roughly trial 71000, at which point it starts switching back and forth between six and seven. This continues until roughly trial 96000, at which point the pool size stays at six up to the 100000^
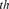
^ trial.

## Discussion

We have explored several closely-related models of pooled screening, allowing for both synergy and antagonism between factors. A basic finding is that pooled testing can have significant benefits in terms of efficiency compared to naive one-at-a-time testing of factors or exhaustive combinatorial testing, as has been found in many other studies (see Hughes-Oliver [14], [26], [27] for a review). This is true even with such impediments as high levels of potential antagonism, noisy readings, or uncertainty in key problem parameters. We derived formulae for the theoretically optimal choice of pool size, showing, to our surprise, that it is unaffected by noise in the testing procedure—although noise does necessitate follow-up testing to confirm results. We also argued that, in the case of noisy testing, it is better not to test every pool in replicate, but only to retest the positive pools. This means that some truly positive pools may pass by as false negatives. Nevertheless, the testing effort involved in being certain about the many truly negative pools is not justified in a high-throughput screening scenario. It is better to wait for a subsequent pool to test positive.

Throughout the paper, we assumed that the pool size should be chosen to minimize the testing cost of phase one—the discovery of a positive pool—and ignored the testing cost of phase two—identification of the synergistic combination from the positive pool. This choice allowed us to derive analytical formulae for the expected number of tests at a fixed pool size and for the optimal pool size. This choice is also justified because in many cases the phase one cost greatly exceeds the phase two costs. However, this is not always the case, especially when the number of blockers is small. For instance, consider searching for a single active compound in a library of 100 factors, only one of which is a blocker. By our memoryless design, the optimal pool size is 50, and allows identification of a positive pool in 3.96 expected tests. By information-theoretic reasoning, subsequently identifying the responsible factor must involve at least 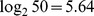 tests. Thus, the second phase has higher expected testing cost than the first phase. A more complete approach would consider the costs in both phases. Although explicit formulae might not be possible to derive, numerical computations could readily be performed to determine the optimal pool size.

We have also assumed that the goal of screening is to identify a single 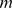-way synergistic combination. Our results can be extended to discovering a fixed number 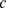 of such combinations by running the proposed scheme repeatedly, until the 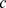 distinct combinations are discovered. We omit a detailed analysis. Intuitively, for a given pool size, each randomly sampled pool would have 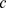 times the chance of being positive as would be the case with a single synergistic combination. Thus, the first combination would be discovered 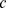 times as quickly as in the case of a single combination. The second combination must be one of the 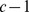 remaining combinations, and so would happen 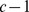 times as quickly; and so on. The total expected number tests would thus be approximately a factor of 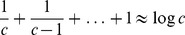 larger than the number of tests required for discovering a single combination. The optimal pool size should not change, because it should be chosen to maximize the probability that any individual randomly-chosen pool is a true positive, regardless of how many synergistic groups there are.

In the memoryless randomized policies we have studied, the pool size is part of the experimental design, but the actual factors chosen to constitute each pool are not—they are simply drawn uniformly randomly from the set of factors. As shown in Remlinger *et al.* [13], for example, biasing this random choice with prior information about the factors can increase the success of screening. In that paper, synergistic effects were modeled but treated as undesirable—a false positive reading. Nevertheless, it would be interesting to try to incorporate some notion of biased random sampling into our analyses, to see if results with similar flavor hold. For that matter, simply keeping track of which pools have already been tested, and favoring pools that have not yet been tested, would likely reduce expected testing effort.

The efficacy of pooling depends strongly on the number of blockers—a parameter which we expect would often be hard to know or even estimate a priori. We showed that even if this parameter is unknown, one can adopt a Bayesian view and maintain a belief state over the number of blockers, which is updated as testing proceeds. The pool size can then be chosen based on this belief. Of course, if one's belief does not match reality at all, poor performance—worse than choosing the minimal pool size—is possible. However, we showed through numerical calculations that for a wide range of the unknown number of blockers, a Bayesian choice of pool size can greatly outperform choosing the minimal pool size.

We also showed that the size of the synergistic combination, 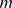, if unknown, can be treated in a Bayesian manner. The procedure we described could also be extended to account for testing errors and even unknown error rates, by maintaining a belief over the error rate parameter. A much greater extension would be to treat the identities of the desirable and blocking factors themselves in a Bayesian manner. If we dispense with the 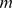 and 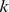 parameters, and imagine that each factor is either desirable, neutral, or blocking, then there are 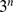 possible ground truths. In principle, we can imagine maintaining beliefs over these 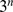 possibilities, and using techniques from the theory of partially observable Markov decision processes [49] to determine an optimal screening strategy. Exact methods would likely be too computationally intensive to apply to this problem, but it would be interesting to see if approximate methods could be applied to generate superior screening designs.

Although genetic and drug interactions were the primary motivations for our work, other application areas could be addressed by the same ideas. While we have already mentioned yeast two-hybrid screening for protein-protein interactions [24], [29], yeast one-hybrid screening for protein-DNA interactions [50] might benefit similarly. Likewise, looking for cancer therapeutics based on small interfering RNAs [51] might benefit from pooling, under the assumption that multiple interfering RNAs are needed to down-regulate the multiple pathways that become mis-regulated in cancer. Recombinant congenic experiments, in which genetically healthy and genetically diseased animals are first cross-bred and then inbred to isolate complex genetic causes of disease, also have a strong flavor of pooled screening [52]. Some of the ideas in this paper might provide novel and useful views of these other types of screening procedures.

## Materials and Methods

### Feasibility of 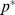


In the first part of our results, where we analyzed a noise-free model of the screening problem, we claimed that the formula for 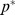, namely 
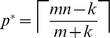
, always produces a value in the range 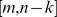. In the case 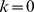, this is immediately true. Otherwise, for 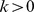, the following shows that 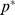 is always at least as large as 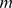:
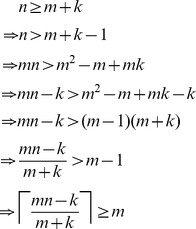



On the other hand, we can see that 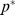 is no larger than 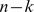 as follows:
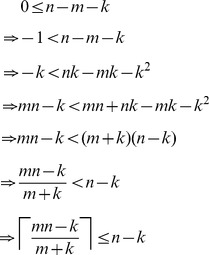



### Probability of confirming a positive test in the noisy model

In our analysis of screening with noisy testing, we claimed that the pool size 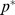 that maximizes the probability of choosing a truly positive test, 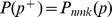, also maximizes the probability of a test being confirmed as positive under the noisy testing model, 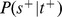. Here, we prove that claim.
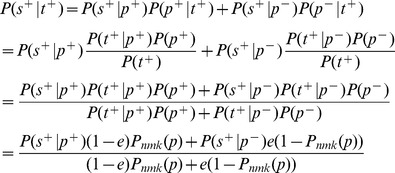
(13)


In the formula above, 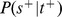 depends on the pool size, 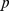, only through the 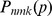 terms. Although it may not be immediately obvious, this formula is increasing as a function of 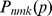. To show this, replace 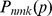 with the variable 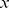 in Equation 13, obtaining:
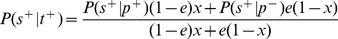
(14)


We treat 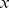 as a real variable in the range 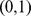, and differentiate with respect to it.
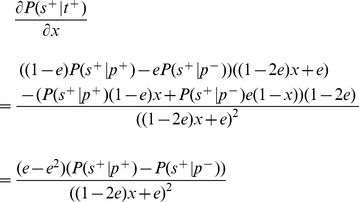
(15)


Because 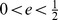, we have 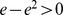. So, the first factor in the numerator is positive. 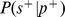 is the probability of obtaining at least 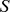 successes out of 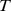 tries, where each try succeeds with probability 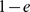. 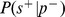 is the probability of at least 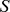 successes when the probability of each success is only 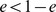. Thus, the former is larger, and the second factor in the numerator above is also positive. This means that 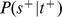 is strictly increasing as a function of 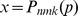. Therefore, choosing 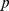 that maximizes 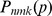 also maximizes 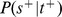.
